# Spatial immune profiling reveals distinct microenvironments in medullary thyroid carcinoma

**DOI:** 10.3389/fimmu.2025.1579205

**Published:** 2025-05-21

**Authors:** Maria Eduarda de Castro, Gustavo Forlin de Siqueira, Jean Ferrante Mariano, Marina Malta Letro Kizys, Lucieli Ceolin, Fernando Augusto Soares, Rodrigo Natal, Humberto Carneiro, Rodrigo Nalio Ramos, Laura Sterian Ward, Niels Olsen Saraiva Camara, Cleber Pinto Camacho, Flavia de Oliveira Facuri Valente, Susan Chow Lindsey, Diego Dias dos Santos, Cristiane Damas Gil, João Roberto Maciel Martins, Rui Monteiro de Barros Maciel, Lucas Leite Cunha

**Affiliations:** ^1^ Laboratory of Molecular and Translational Endocrinology, Department of Medicine, Escola Paulista de Medicina, Universidade Federal de São Paulo, São Paulo, Brazil; ^2^ Pathology Division, D’Or Research Institute, Rede D’Or Hospitals Network, São Paulo, Brazil; ^3^ Department of Immunology, Institute of Biomedical Sciences, University of São Paulo, (USP), São Paulo, Brazil; ^4^ Laboratory of Cancer Molecular Genetics, School of Medical Sciences, University of Campinas (UNICAMP), Campinas, São Paulo, Brazil; ^5^ Molecular Innovation and Biotechnology Laboratory, Postgraduate Medicine Program, Universidade Nove de Julho (UNINOVE), São Paulo, Brazil; ^6^ Department of Morphology and Genetics, Escola Paulista de Medicina (EPM), Universidade Federal de São Paulo (UNIFESP), SP, São Paulo, Brazil; ^7^ Emergency Medicine and Evidence Based Medicine Unit, Department of Medicine, Escola Paulista de Medicina, Universidade Federal de São Paulo, São Paulo, Brazil

**Keywords:** medullary thyroid carcinoma (MTC), immune microenvironment, PD-L1 expression, tumor-infiltrating lymphocytes (TILs), mast cells

## Abstract

**Introduction:**

Medullary thyroid carcinoma (MTC) is a rare and aggressive thyroid cancer with a challenging prognosis. While the immune microenvironment plays a crucial role in cancer progression, its role in MTC remains underexplored compared to more common thyroid cancers.

**Methods:**

this study investigates the immune landscape of MTC by systematically evaluating immune cell infiltration and expression of immune markers across various tissue topographies. We utilized advanced immunohistochemical techniques to analyze tissue samples from 24 MTC patients, focusing on the tumor core, interface with healthy tissue, adjacent normal thyroid tissue, and lymph node metastases.

**Results:**

our findings reveal a distinct immune profile with increased CD3+, CD4+, CD8+ and CD20+ lymphocytes in normal tissues adjacent to tumors and a notable presence of granzyme B+ cells in the tumor interface, particularly in patients with structural disease. Additionally, we observed a significant enrichment of mast cells in metastatic tissues.

**Discussion:**

these results highlight the complex and spatially dependent immune landscape of MTC, suggesting implications for targeted immunotherapy. This study provides novel insights into the immune microenvironment of MTC and emphasizes the need for further research to elucidate its impact on disease progression and therapeutic response.

## Introduction

Medullary thyroid carcinoma (MTC) is a rare yet aggressive form of thyroid cancer arising from parafollicular C cells (1, 2). Clinically, it is characterized by thyroid nodule and elevated calcitonin levels (3). Despite advances in diagnosis and treatment, the prognosis of MTC remains challenging, with variable survival rates and frequent recurrence, even after surgery and adjuvant therapies (4). The heterogeneous prognosis among patients underscores the urgent need for more precise predictive tools to stratify risks and guide personalized therapeutic decisions.

The immunological microenvironment plays a crucial role in the progression of thyroid cancer, particularly evident in papillary thyroid carcinoma (PTC), the most common type (5). Studies have revealed a complex interplay between tumor cells and components of the immune system, including tumor-infiltrating lymphocytes (TILs) and tumor-associated macrophages (TAM) (6). These interactions influence not only the initial treatment response but also the propensity for metastasis and therapeutic resistance (7).

Nonetheless, studies on the immunological microenvironment of MTC are limited. Previous research suggests a significant presence of TILs and regulatory T cells (Tregs) in the tumor microenvironment of MTC, potentially affecting disease progression and treatment response (8). Furthermore, the expression of immune molecules, such as PD-L1, has been associated with immune evasion in MTC, highlighting its potential as a therapeutic target (9). A comprehensive understanding of these immunological mechanisms is crucial for a better prognostic definition and the development of novel therapeutic strategies for MTC.

Several prognostic markers and treatment protocols have been established and are currently being employed (9, 10). However, comprehensive data integrating histopathological, immunohistochemical, and molecular analyses to understand the tumor microenvironment in MTC, especially with regard to immune cell infiltration and its clinical implications, remain limited. We aimed to fill this gap by systematically evaluating the immune landscape of the MTC tissues. Our objectives were to quantify immune cell infiltration in different tissue regions, evaluate the expression of immune checkpoints, and correlate these findings with the clinical outcomes.

## Materials and methods

### Design

This study was approved by the Research Ethics Committee of Hospital São Paulo and Universidade Federal de São Paulo, São Paulo, Brazil (309–2023). We investigated 24 patients of the BRASMEN project (CAAE: 70952523.8.0000.5505) with histopathological confirmation of MTC, whose clinical data were updated from data collected from their medical records (11). Aggressiveness at diagnosis was determined using the tumor-node-metastasis classification and staging system for MTC (11). All patients followed a treatment protocol established by the American Thyroid Association (4).

The tissues of the patients, fixed in formalin and embedded in paraffin, were reviewed for diagnostic confirmation and selection of the most representative areas for the construction of a Tissue Microarray (TMA) (Beecher Instruments, Silver Springs, MD, USA). From each case, two tissue samples with an estimated area of ​​0.79 mm² were obtained from the following regions: (i) tumor core, (ii) tumor interface with healthy thyroid tissue, (iii) adjacent healthy thyroid tissue, and (iv) lymph node metastasis (when present).

The inclusion criteria were as follows: (i) diagnosis of MTC in any age group, (ii) clinical data available for evaluation, (iii) primary tumor tissues stored in paraffin available for morphological investigation, and (iv) primary tumor tissues stored in paraffin with quality and viability for molecular studies. Patients were excluded if: (i) they did not have clinical data in their medical records; (ii) they did not present tissue samples in a pathological anatomy repository; (iii) the diagnosis of MTC could not be confirmed by histological slide review; (iv) molecular diagnosis of MEN2 could not be performed by sequencing the RET gene and; (v) the paraffin tissue was not of sufficient quality for molecular analysis.

After total thyroidectomy, patients were classified as: i) biochemical disease-free, in other words, no laboratory evidence of residual disease or recurrence when measuring serum calcitonin or serum carcinoembryonic antigen (CEA); ii) structural disease-free, when there was no evidence of visible disease on imaging tests such as PET-CT; iii) disease-free, those patients who have neither biochemical nor structural disease.

### Immunohistochemistry

**Figure 1:**
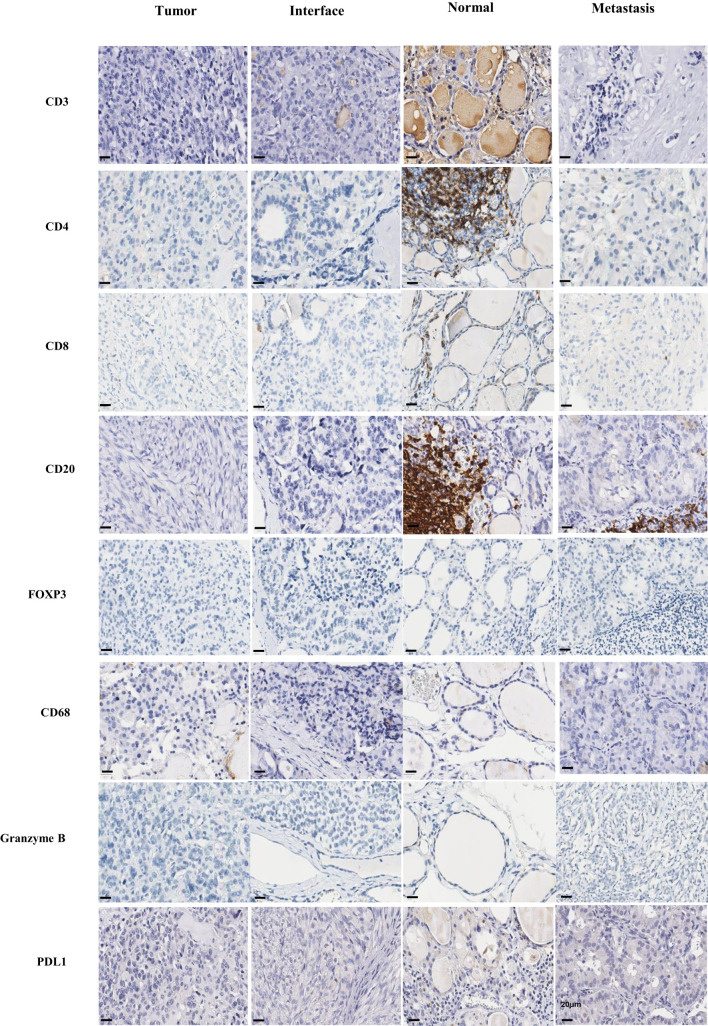
Immunohistochemical. Immunohistochemistry was carried out in the tumor, interface, normal tissue adjacent to the tumor and metastasis regions for the markers CD3, CD4, CD8, CD20, FOXP3, CD68, granzyme B and PDL1. Scale bar, 20 µm, identified in the bottom left corner of each image.

Immunohistochemistry was performed in a hospital clinical routine laboratory, whose immune cell markers analyzed included TAM (CD68) and subsets of TIL, such as CD3, CD4, CD8, and CD20. The activation status of these immune cells was identified using Granzyme-B and PD-L1(22C3) (**Figure 1**).

The analyses were carried out by immunohistochemical staining using the ultraview universal DAB detection kit ventana (Roche) and EnVision FLEX Visualization Systen kit AGILENT (DAKO), according to standard operating protocols. The corresponding positive and negative controls were analyzed and found to be adequate. The respective antibodies are: rabbit polyclonal anti-CD3 antibody (DAKO); mouse monoclonal anti-human anti-CD20 (L26 - DAKO); rabbit monoclonal anti-FOX P3 (EP340 - BIOSB); rabbit monoclonal anti-CD4 (SP35 - Ventana); rabbit monoclonal anti-CD8 (SP57 - Ventana); mouse monoclonal anti-CD68 (PG-M1 - DAKO); mouse monoclonal anti-PD-L1 (22C3 - DAKO); rabbit polyclonal anti-granzyme B (Roche).

The independent assessment of immune cell markers was carried out by calculating the count of positive cells per TMA point based on an estimated area of ​​approximately 0.79 mm². The cases were grouped into the following categories for statistical analysis: 0 (complete absence of immune cells), 1 (up to ten immune cells), and 2 (more than 10 immune cells). Visual assessment of PD-L1(22C3) was performed by estimating the percentage of positive cells according to the Combined Positive Score (CPS), which is defined as the number of PD-L1 positive cells divided by the total number of tumor cells × 100. The PD-L1 CPS cutoffs were: <1 (negative) and ≥ 1 (positive). Since only 22C3 clone was positive in the samples, only 22C3 clone was considered for further analysis.

We also assessed the presence of tumor necrosis and quantified mitotic activity and Ki67 expression. Tumor necrosis was evaluated visually on hematoxylin and eosin-stained slides and classified as either present or absent. Mitotic activity was quantified by counting mitotic figures in ten high-power fields (HPFs, 40× objective) within the most mitotically active area of the tumor, and the results expressed as the number of mitosis per 10 HPFs. Ki67 expression was analyzed by immunohistochemistry using a mouse monoclonal anti-Ki67 antibody (MIB-1; Dako, Glostrup, Denmark). The Ki67 labeling index was calculated as the percentage of positively stained tumor nuclei in a total count of at least 1,000 cells in the area of the highest labeling density (“hot spot”) identified at low magnification (10×).

The tumors were classified as low, intermediate, or high based on the mitotic count, ki-67 proliferative index, and presence or absence of necrosis. Low proliferative activity was defined as <3 mitoses/10 HPFs, a Ki-67 proliferative index of <3%, and absence of coagulative necrosis. An intermediate degree of the proliferative index was considered when there were 3 to 20 mitoses/10 HPFs or a Ki-67 index of 3% to 20%, without coagulative necrosis. Cases with <3 mitoses/10 HPFs and a Ki-67 proliferative index of <3%, but with coagulative necrosis were also considered intermediate grade. A high-grade proliferative index was considered when there were 3–20 mitoses/10 HPFs2 or a Ki-67 index of 3–20% and coagulative necrosis. Cases with >20 mitoses/10 HPFs or a Ki-67 index of >20% were also considered high-grade, regardless of whether or not there was coagulative necrosis.

QuPath software made it possible to quantify the number of positively labeled cells over the total number of cells, in the entire region of the section (12). Furthermore, to standardize the area evaluated, we estimated the value of positive cells in the area of ​​1 mm² and this was the number used for subsequent statistical analysis.

We conducted a hierarchical cluster analysis of our immunohistochemical data using Cluster 3.0 software to evaluate the similarities and distances between patients based on the immunological parameters. In accordance with the authors’ guidelines, we applied complete-linkage hierarchical clustering to the following eight parameters: CD3, CD20, FOXP3, CD4, CD8, CD68, PDL1 (22C3 clone), Granzyme B, and mast cells. The resulting dendrogram, which visually represents patient similarities and distances, was generated using Java TreeView software (13).

### Assessment of mast cell infiltration

Mast cell infiltration was assessed using a Rapid Panotic technique (Larboclin). Sections obtained from the samples were prepared as described in the immunohistochemistry section. To visualize mast cells, the slide was immersed for 1 min in solution I (0.1% triarylmethane), 1 min in solution II (0.1% xanthenes), and 30 s in solution III (thiazines to 0.1%). Sections were washed in water buffered at pH 7 and dehydrated for slide preparation. Mast cells were identified and characterized by visualization of metachromatic cytoplasmic granules. The cells were quantified using the 40x objective of QuPath software in the total area of ​​the sections, approximately 0.79 mm².

### Statistical analysis

All slides were scanned by ScanScope CS² using a 40x objective for subsequent quantification of immunolabeling using the QuPath software. Clinical, anatomopathological, and immunohistochemical data were crossed in search of associations with statistical significance using the chi-square method for quantitative variables and the Kruskal-Wallis test for independent samples to perform non-parametric tests for ordinal and numeric variables. GraphPad software (version 9) was used to present data as the mean ± standard error of the mean (S.E.M.). Non-normally distributed data were used for datasets with more than two groups. Statistical analysis was performed using the Statistical Package for the Social Sciences (SPSS, Chicago, IL, USA)(R) software, version 23.0. Statistical significance was set at P < 0.05. We performed Benjamini-Hochberg correction to reduce the bias caused by multiple testing, adjusting the p-value to reduce the false discovery rate. All adjusted p-values (Q) < 0.05 were considered significant.

## Results

### Patient characteristics

**Table 1** shows the demographic, clinical, and pathological characteristics of the patients. The majority of patients were diagnosed after the age of 45 years and were female. Most included patients had sporadic MTC. **Table 2** summarizes the main pathological characteristics of the investigated tumors. The most common histological type was the classic type, with 62.5% of patients presenting with multifocality, surgical margins free in less than 50% of cases, and a minority with tumors larger than 4 cm. Thyroiditis was present in 12.5% of the cases, and TNM staging was well distributed. Central lymph node invasion was observed in 52.6% of patients. The patients were followed-up in our service for 10 years. Most patients had structural or biochemical remission of the disease after surgery; however, only 36.4% were free of concomitant biochemical and structural disease after surgery (**Table 1**).

**Table 1:** Descriptive table of demographic and clinical characteristics.

Variable	Category	N	%
Gender	Female	14	58.3
	Male	10	41.7
Age	≤45 years old	6	25
	>45 years old	18	75
Syndrome	Sporadic	17	70.8
	*MEN 2*	7	29.2
Relapse-free survival	Yes	8	36.4
	No	14	63.6
Structural disease	Yes	6	27.3
	No	16	72.7
Biochemical disease	Yes	8	36.4
	No	14	63.6

**Table 2:** Descriptive table of anatomopathological features.

Variable	Category	N	%
Histological MTC type	Classic	18	75
	Non-classic	6	25
Multifocality	Absent	9	37.5
	Present	15	62.5
Surgical margins	Free	11	45.8
	*Exiguous*	7	29.2
	Compromised	6	25
Tumor size	<2 cm	11	45.8
	2–4 cm	10	41.7
	>4 cm	3	12.5
Thyroiditis	Absent	21	87.5
	Present	3	12.5
TNM (cT)	1	10	41.7
	2	8	33.3
	3	4	16.7
	4	2	8.3
TNM (cN)	0	5	23.8
	1	8	38.1
	2	8	38.1
TNM (cM)	0	4	16.7
	1	7	29.2
	X	13	54.2
Central Lymph node Invasion	Absent	9	47.4
	Present	10	52.6

Tumor necrosis was not observed in any of the cases. Mitotic activity varied greatly from 0 to 4 mitoses per 10 high power fields (HPFs), with a median of 0 mitoses/10 HPFs. A high mitotic index (>3 mitoses/10 HPFs) was present in 12.5% of the cases. The Ki67 labeling index ranged from 0% to 5% and the proliferative index was considered low in 95.8% of cases.

### Semi-quantitative analysis of immunological markers

**Supplementary Table 1** shows the results of the semi-quantitative analysis of the infiltration of different immune cells in various histological topographies. We did not observe any difference in the categories of immune cell infiltration among samples from different topographies. PD-L1 positivity was rare and equally scarce across the different tissues evaluated.

### Quantitative analysis of immunological markers

**Figure 2:**
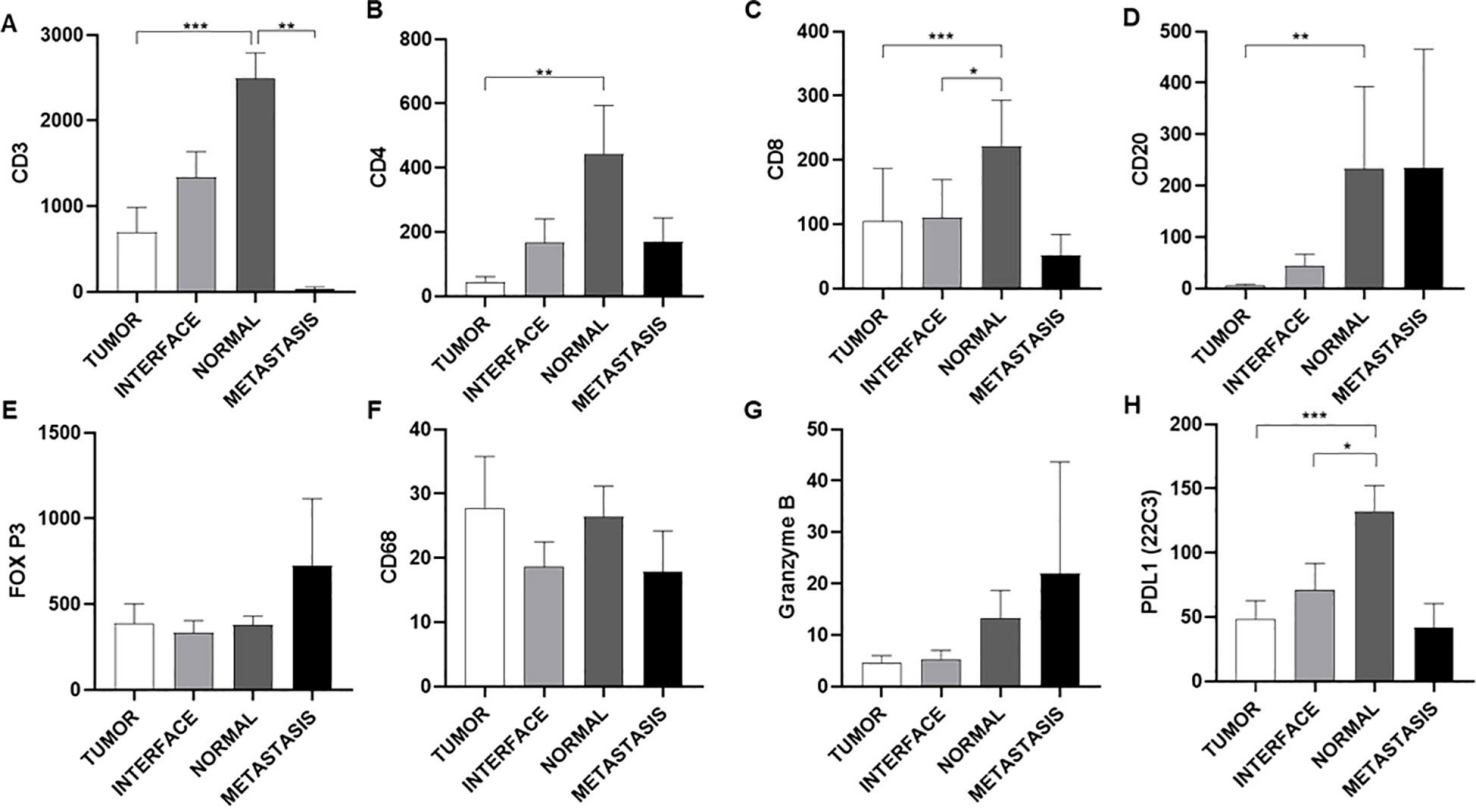
Quantitative analysis of immune cell infiltration and expression of immunological markers in different regions of tumor tissue. Quantification of lymphocyte and macrophage markers **(A-H)**. The analysis reveals a general decrease in the infiltration of T lymphocytes (CD3, CD4, CD8) and B cells (CD20) in tumor and metastatic tissue compared to normal tissue, suggesting an immunosuppressive tumor microenvironment. The low expression of PD-L1 in the tumor shows that this is not one of the main mechanisms of immune escape. The Y-axis indicates the marker analyzed, while the X-axis shows the region analyzed. Data are presented as mean ± S.E.M of protein expression in positive cells per mm², and dot plots represent individual values per group (n = 24 patients/group). *P < 0.05; **P < 0.01; ***P < 0.001 vs. Normal (Kruskal-Wallis, Dunn’s post-test).

In general, normal thyroid tissue samples adjacent to the tumor were enriched with immune cells of different immunophenotypes. Immune cell infiltration was analyzed using immunohistochemistry and semi-automatic quantification. Normal tissue samples adjacent to the tumors showed greater infiltration of CD3+ lymphocytes than the center of the tumor (p<0.001) and the region of metastasis (p<0.01; **Figure 2A**). Normal tissues adjacent to the tumor (p<0.01) showed greater infiltration of CD4+ lymphocytes than samples from the tumor center (**Figure 2B**). Normal thyroid tissues adjacent to the tumor were more enriched with CD8+ lymphocytes than the tumor center (p<0.001) and interface (p<0.05; **Figure 2C**). Normal thyroid tissues showed greater infiltration of CD20+ lymphocytes than samples from the tumor center (p<0.01; **Figure 2D**). Metastatic tissue samples were enriched with immune cells of different immunophenotypes, although statistical analysis showed no differences between other topographies (**Figures 2B–H**). The samples exhibited sparse infiltration of granzyme B+ lymphocytes, with a tendency towards greater infiltration in metastatic tissues (**Figure 2G**). Immunohistochemical evaluation of PD-L1 was positive for this marker in all tissue topographies analyzed, with clone 22C3 showing a higher number of positive cells per mm² in the normal tissue adjacent to the tumor when compared to the tumor center (p < 0.001) and the interface region (p < 0.05) (**Figure 2H**).

**Figure 3:**
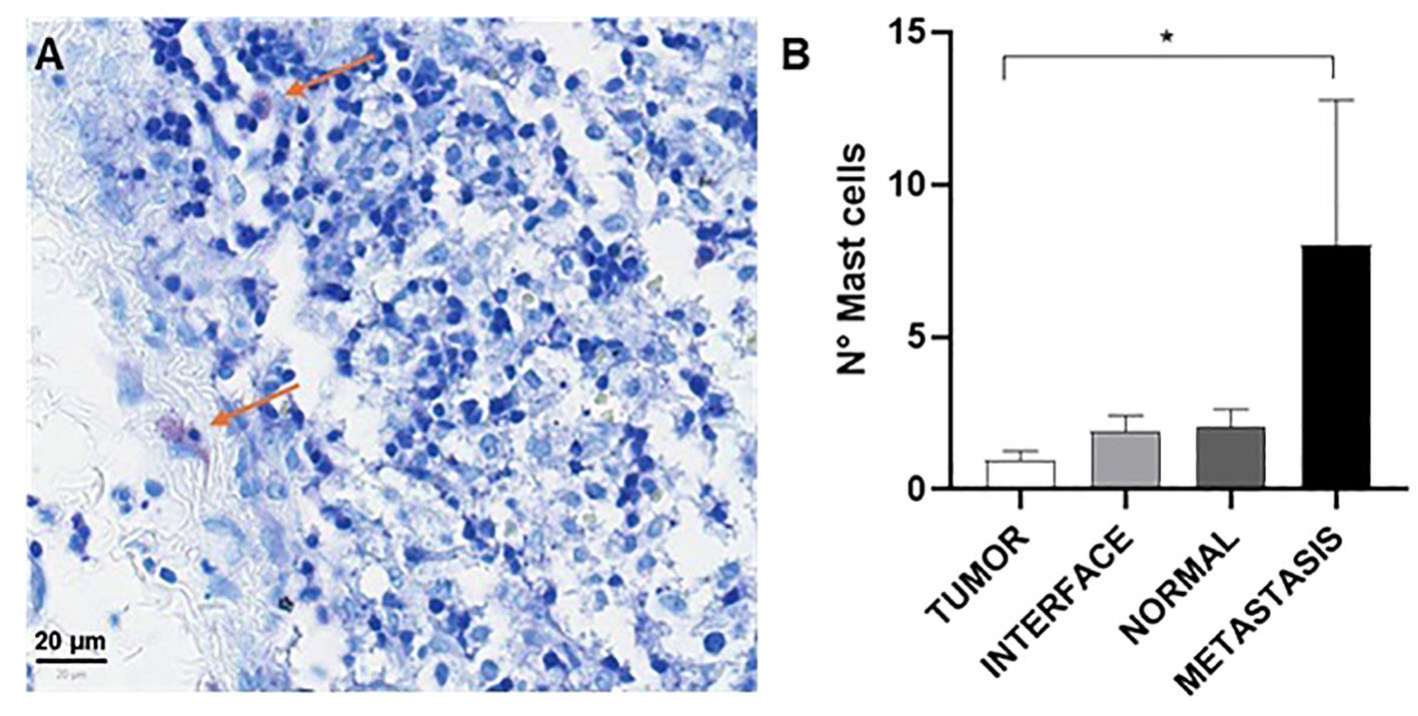
Mast cell analysis. Representative image of histological staining, showing mast cells identified by their characteristic purple staining indicated by red arrows **(A)**. Quantitative analysis revealed a significant increase in the number of mast cells in the region of metastasis compared to the other regions, suggesting a possible association between the presence of mast cells and the metastatic process. Quantification of mast cells per 0.79 mm² **(B)**. Data are represented as mean ± standard error of the mean (S.E.M) of the number of cells per 0.79 mm², and dot plots show individual values by group (n = 24 patients/group). *P < 0.05 (Kruskal-Wallis, Dunn’s post-test).

Analysis of tissues using rapid panoptic staining revealed the presence of a few mast cells across different histological topographies (**Figure 3A**). Metastatic tissue samples showed greater infiltration of these cells compared to other topographies, especially when compared to the tumor center (p<0,05; **Figure 3B**).

### Analysis of clinical status and clinical characteristics

**Supplementary Table 2** summarizes the clinical description of the patients included in this study. Patients with T1 tumors (tumor limited to the thyroid of 2 cm or less) were predominantly found in the group without evidence of active disease (62.5%), followed by those with biochemical disease (37.5%), and none in the group with structural disease (P=0.366). The distribution of T2 tumors (tumor limited to the thyroid larger than 2 cm and smaller than 4 cm) was 25% without active disease, 37.5% with biochemical disease, and 37.5% with structural disease. T3 tumors (tumor with extrathyroidal extension or larger than 4 cm limited to the thyroid) accounted for 25% of patients without active disease, 25% with biochemical disease, and 50% with structural disease. T4 tumors (moderately or very advanced disease, invading other tissues) showed 50% with biochemical disease and 50% with structural disease.

In terms of nodal status, N0 patients (no metastases in regional lymph nodes) were mainly in the group with no evidence of active disease (100%), and none with biochemical or structural disease (P=0.004). N1 patients (metastases in lymph nodes at level VI) were distributed as follows: 28.6% without active disease, 57.1% with biochemical disease, and 14.3% with structural disease. N2 patients (lymph node metastases to other levels) showed 0% without active disease, 50% with biochemical disease, and 50% with structural disease.

Patients with M0 status (no distant metastasis) were predominantly without evidence of active disease (66.7%) and some had biochemical disease (33.3%), with no evidence of structural disease (P=0.230). M1 status (distant metastasis) patients were more distributed: 14,3% without active disease, 28,6% with biochemical disease, and 57,1% with structural disease. Patients with Mx status (it was not possible to identify) were more distributed: 37.5% without active disease, 43.8% with biochemical disease, and 18.8% with structural disease.

The absence of multifocality was associated with 66.7% of patients without evidence of active disease, 22.2% with biochemical disease, and 11.1% with structural disease (P=0.046). The presence of multifocality was associated with 15.4% of patients without active disease, 46.2% with biochemical disease, and 38.5% with structural disease.

Patients who underwent total thyroidectomy (TT) without central emptying had 66.7% without active disease and 33.3% with biochemical disease (P=0.092). Those who had TT with central emptying comprised 57.1% without active disease, 42.9% with biochemical disease, and none with structural disease. Patients with TT with central and lateral emptying showed 16.7% without active disease, 33.3% with biochemical disease, and 50% with structural disease.

### Analysis of immunological markers versus pathological and clinical characteristics

Mitotic activity showed a positive relationship with CD68+ infiltration in the metastasis region (Spearman = 0.470; P = 0.032) and a positive relationship with CD4+ (Spearman = 0.949; P = 0.051) and CD8+ (Spearman = 0.949; P = 0.051) infiltration as well as in the metastasis region, but with a non-significant marginal p-value. Tumor size was negatively correlated with infiltration and CD3+ cells in the tumor region (Spearman = -0.497; P = 0.022). Ki-67 expression in the interface region showed a negative relationship with CD68+ infiltration in the normal tissue region adjacent to the tumor (Spearman = -0.458; P = 0.042).

**Figure 4:**
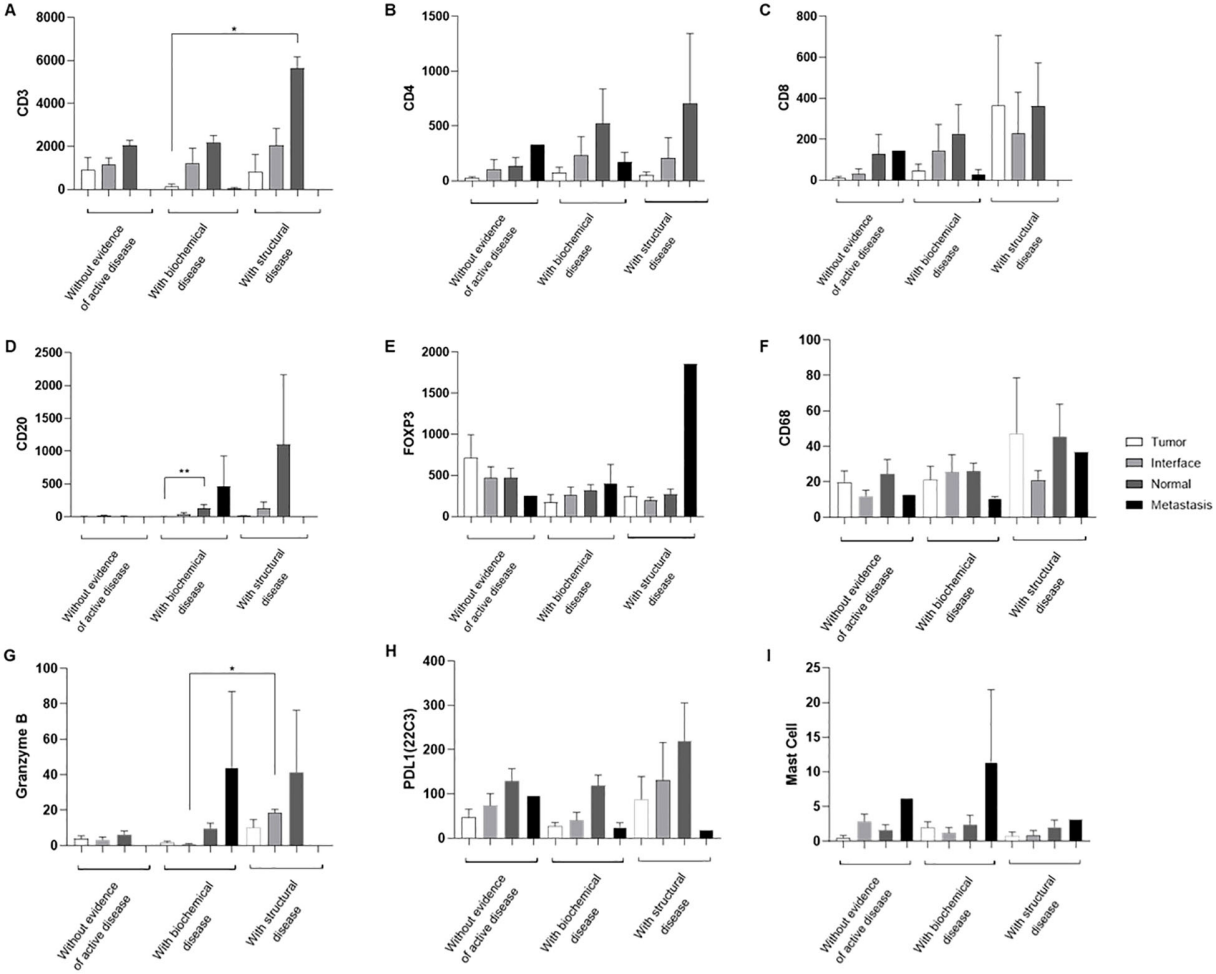
Marker analysis according to clinical status. Cross-analysis between cell expression (Y-axis) and patient clinical status (X-axis), including no evidence of active disease, biochemical disease, and structural disease **(A-I)**. The legend indicates the analyzed tissue regions. Data are shown as means ± S.E.M of protein expression in positive cells per mm², and dot plots represent individual values by group (n = 24 patients/group). *P < 0.05; **P < 0.01 vs Tumor (Kruskal-Wallis, Dunn’s post-test).

Patients were classified according to their clinical status as follows: (i) no evidence of active disease, (ii) biochemical disease, or (iii) structural disease. Following this classification, we analyzed the infiltration of immune cells according to each status. Normal thyroid tissue showed greater infiltration of CD3 cells, especially in individuals with structural disease (**Figure 4A**). In addition, metastatic tissues showed a sparse infiltration of CD3, CD4 and CD8 cells in all clinical statuses (**Figures 4A–C**). Patients with biochemical disease presented with normal thyroid tissue with a higher infiltration of CD20 cells than the tumor core (P<0.01). The same trend was observed in patients with structural disease (**Figure 4D**). The expressions of FOXP3 and CD68 were not significant (**Figures 4E, F**). The interface tissue of patients with structural disease showed greater infiltration of granzyme B+ cells than the interface area of individuals with biochemical disease (p<0.05; **Figure 4G**). PDL1 (22C3) showed higher expression in normal tissues adjacent to the tumor, but not significantly (**Figure 4H**). Mast cell showed no statistical relevance or significant trends (**Figure 4I**).

### Analysis of the mixture of immune microenvironment markers

**Figure 5:**
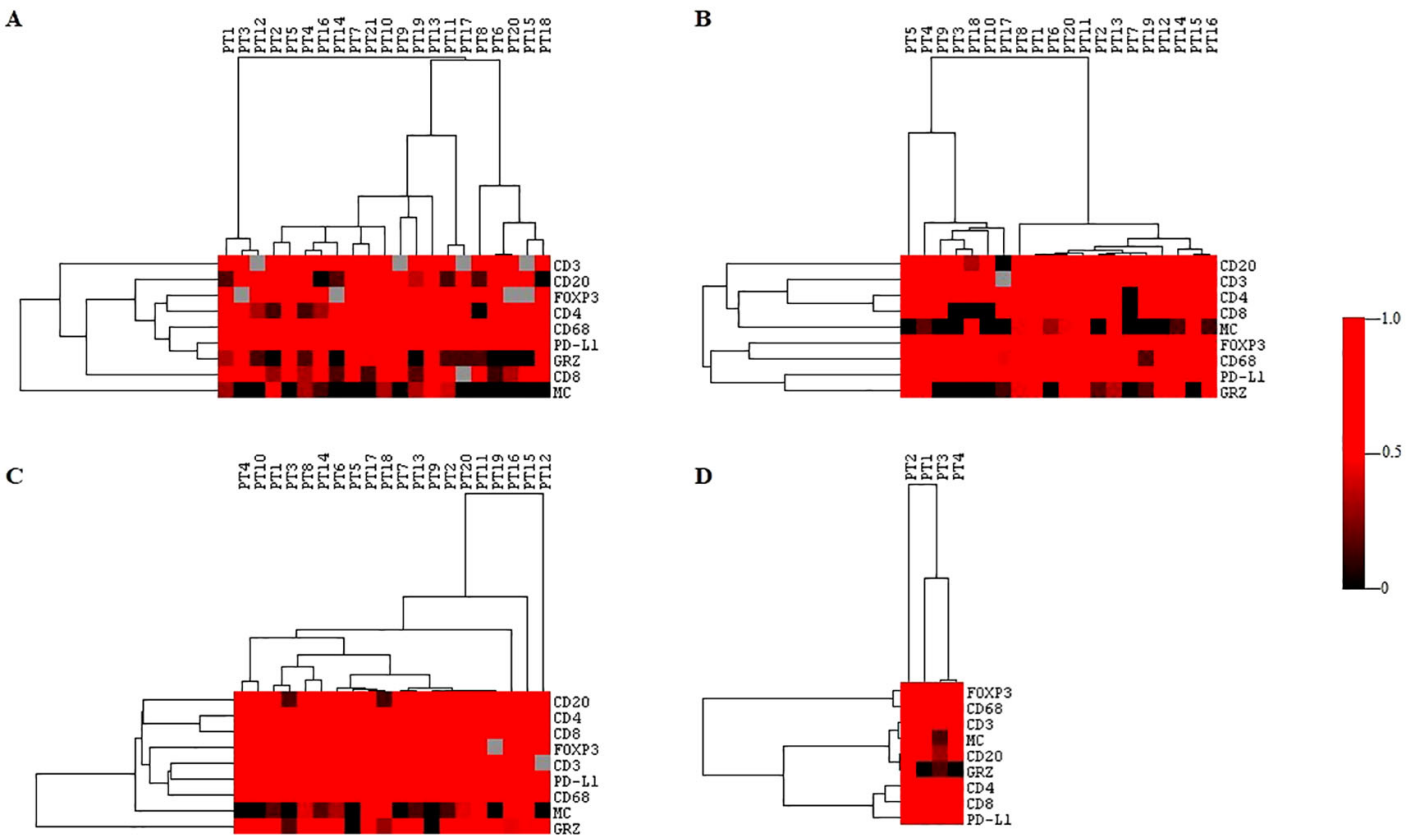
Cluster analysis of the immune microenvironment in different topographies. Hierarchical clustering of immune markers in the four topographies. The immune response profiles were grouped into clusters based on the similarities and differences between the immune markers. **(A)** center of the tumor. **(B)** invasive margin. **(C)** normal thyroid tissue adjacent to the tumor. **(D)** metastasis. Each column represents an individual sample and each row corresponds to a specific immune marker. GRZ, granzyme B; MC, mast cell. The color scale indicates the normalized expression intensity, with red representing high expression and black representing low expression. The greater the intensity of the color, the greater the relationship between the markers.

**Figure 6:**
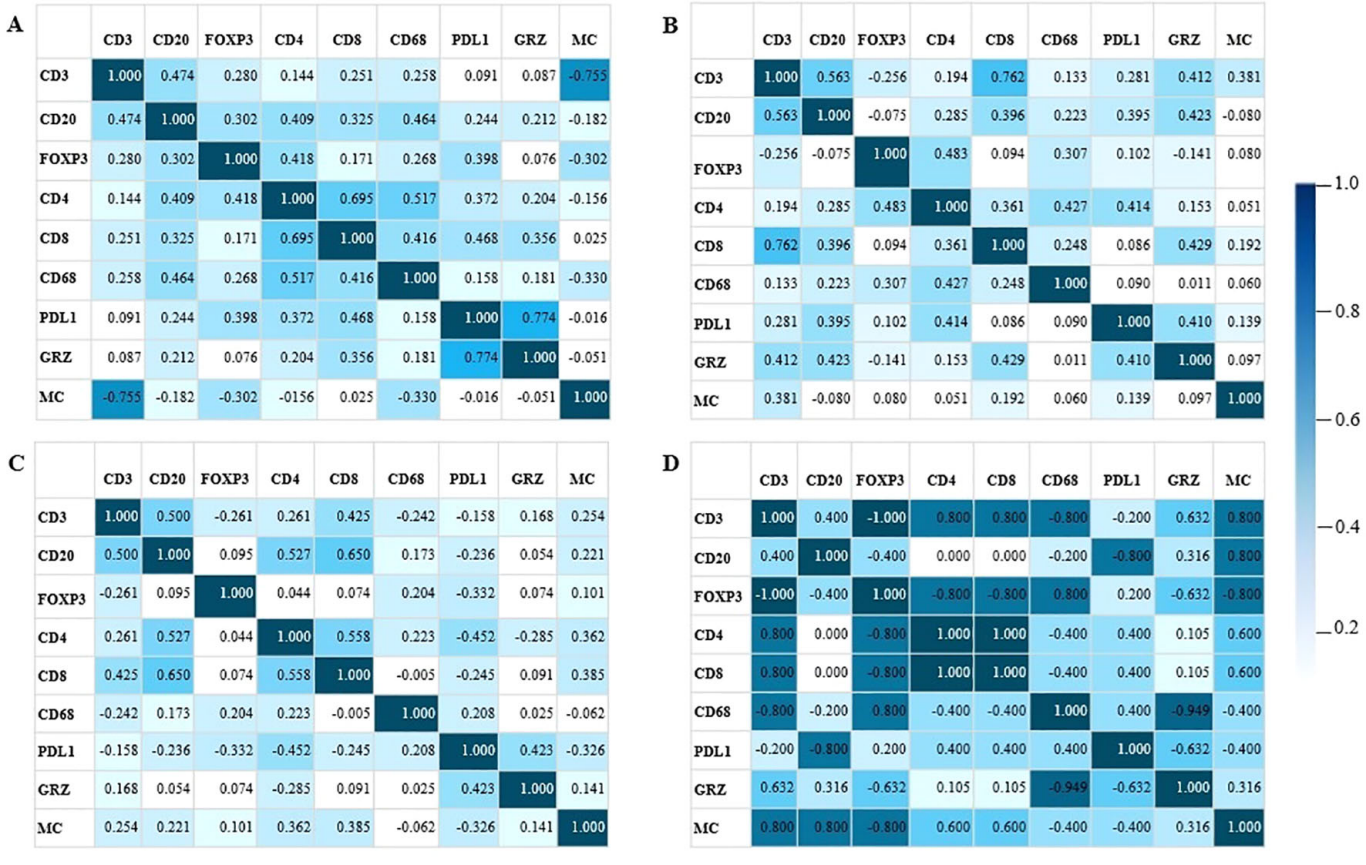
Correlation matrix. The values express Spearman’s correlation coefficients between the density of immunological markers and mast cells (MC) in four different regions of the tumor microenvironment. **(A)** center of the tumor. **(B)** invasive margin. **(C)** normal thyroid tissue adjacent to the tumor. **(D)** metastasis. Positive values indicate a positive relationship between the markers and negative values indicate an inverse relationship. GRZ, granzyme B; MC, mast cell. The bar next to it indicates the intensity of the relationship between the markers. The greater the intensity of the color, the greater the relationship between the markers.

Cluster analysis showed that each histological topography exhibited a unique pattern of similarities and discrepancies among the markers, as shown in **Figure 5**. Furthermore, the correlation matrix analysis revealed that each microenvironment topography presents unique patterns of bivariate correlations between the biomarkers, as visually demonstrated in **Figure 6**.

First, analyzing the microenvironment of the tumor core (**Figure 6A**), the sparse infiltration of mast cells in the tumor core resulted in this marker showing greater discrepancy compared to other immune markers. Immune function markers such as PD-L1 and Granzyme, demonstrated a stronger correlation of positivity within the tumor core. Mast cells, on the other hand, showed an intense negative relationship with CD3.

In the interface region (**Figure 6B**), the profile of immune response markers differed from that of the tumor core by exhibiting two major groups of immune responses that diverged in terms of Granzyme B+ cell infiltration. In addition, CD3 and CD8 showed a strong positive correlation.

In normal tissue adjacent to the tumor (**Figure 6C**), the immune response appeared relatively uniform, with minimal variation in similarities and distinctions among the immune response markers.

The metastasis microenvironment was characterized by strong correlations (**Figure 6D**). In the metastatic microenvironment, we observed a strong inverse correlation between PD-L1 expression and Granzyme B+ lymphocyte infiltration. In this microenvironment, mast cell infiltration is higher than in other topographies and positively correlates with the enrichment of various other immune cells, such as CD3+, CD4+, CD8 and CD20+ lymphocytes. Conversely, mast cell infiltration was inversely correlated with FOXP3, CD68 and PD-L1 expression levels.

We corrected the p-values using the Benjamini-Hochberg test to obtain the corrected p-values (Q). The correlation of the markers in the intratumoral region revealed a positive and significant correlation coefficient (Spearman = 0.774; Q = 0.001) of Granzyme B with PDL1 and CD4 with CD8 (Spearman = 0.695; Q = 0.006). CD3 and mast cells showed a negative relationship (Spearman = -0.755; Q = 0.001). The interface region showed a positive and significant correlation (Spearman = 0.762; Q = 0.005) between CD3 and CD8. While the normal region adjacent to the tumor showed no significant Q.

## Discussion

**Figure 7:**
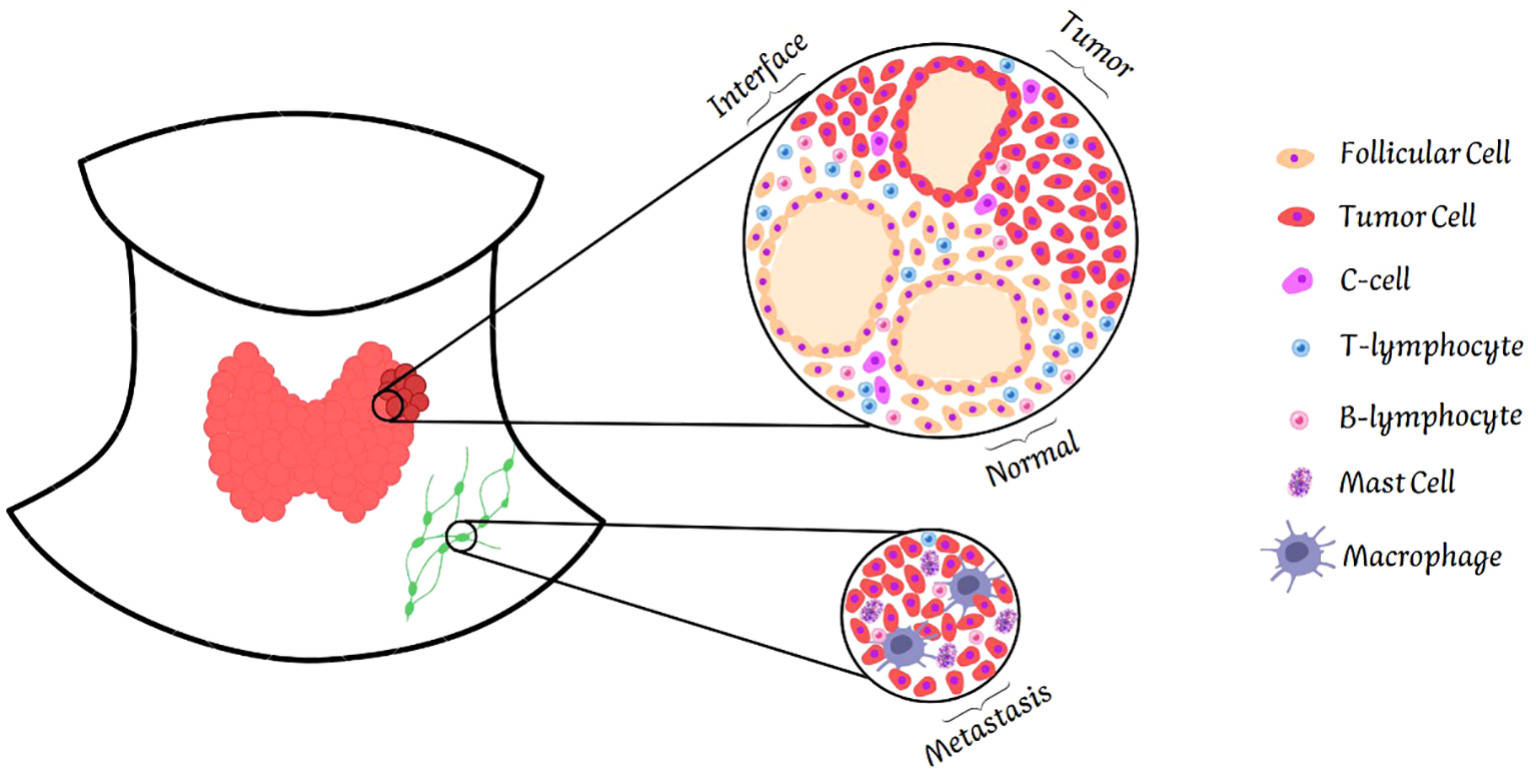
Illustration of the tumor microenvironment. The figure shows the four areas of the tumor analyzed: normal tissue, interface, tumor center and metastasis. Normal tissue shows greater infiltration of T and B lymphocytes. The interface follows the same pattern. In contrast, the tumor center is poor in these cells. Metastasis differs from other tissues, showing infiltration not only of T and B lymphocytes, but also of macrophages and mast cells.

To our knowledge, this is the first study to demonstrate the spatial dependence of the immune microenvironment on the immune profile of MTC and its relationship with the demographic and pathological characteristics of the patients. Immune profiling indicated that normal thyroid tissues adjacent to tumors were enriched with CD3+, CD4+, CD8+, and CD20+ lymphocytes compared with tumor centers. Metastatic tissues exhibited a tendency to greater infiltration of CD20+ immune cells, although this was not consistently significant. The immune profile highlighted that the immune microenvironment of the interface tissue, where normal thyroid tissue meets the tumor, showed a distinct pattern of immune cell infiltration. This region showed a greater infiltration of CD3+ and CD4+ lymphocytes than the center of the tumor, suggesting a robust immune response at the margins of the tumor. In addition, the interface tissue showed greater infiltration of granzyme B+ cells, particularly in patients with structural disease. **Figure 7** illustrates the CMT tumor microenvironment. These results indicate that the interface tissue plays a critical role in the immune response against MTC, with potential implications for prognosis and therapeutic strategies.

The spatial-dependent immune landscape of the inflammatory immune microenvironment in MTC is consistent with findings from other studies, which highlight a complex interaction between immune cells and tumor progression (14, 15). Studies have shown that normal thyroid tissue adjacent to tumors often exhibits a higher density of immune cells, particularly CD3+, CD4+, CD8+, and CD20+ lymphocytes, indicating a robust immune response in non-tumor tissues (7). Similarly, metastatic tissues frequently demonstrate increased infiltration of mast cells and granzyme B+ lymphocytes, reflecting a localized inflammatory response associated with advanced disease stages (16, 17). The distinct clustering patterns of immune markers, such as the correlation of CD4 and CD8 cells with inflammatory responses in the tumor interface, have also been reported in the literature (18, 19), emphasizing the dynamic and heterogeneous nature of the immune microenvironment in MTC. These findings corroborate the notion of an intricate immune landscape within MTC, characterized by diverse immune cell populations and marker expressions, underscoring the potential for targeted immunotherapeutic strategies, as discussed in recent studies.

While the expression of immune checkpoint markers, such as PD-L1, is generally sparse across different tissue topographies, its elevated levels at metastatic sites suggest an immune evasion mechanism, consistent with observations in other cancer types (20, 21). The knowledge about PD-L1 expression in MTC is limited, and the few existing studies are inconsistent, with varying results (9, 22–24). In most of these studies, PD-L1 is minimally expressed in tumor cells, and in one study, its very low expression did not correlate with clinical characteristics (9, 22–24). Our study showed that PD-L1 (clone 22C3) is more highly expressed in normal tissue adjacent to the tumor than intratumoral tissue, revealing that this is not the main mechanism of lymphocyte inactivation in MTC and is therefore not a good way to treat patients. Some studies have similar results to ours; analysis with the SP142 and SP263 clones revealed almost no PD-L1 expression in MTC cells and inflammatory cells, and no clinicopathological or prognostic association (22, 24). A Chinese study with 49 patients analyzed PD-L1 expression for clones 22C3 and SP142; neither clone was significantly expressed in MTC and was not related to clinicopathological parameters (25).

Furthermore, we demonstrated for the first time that mast cells are abundant in the metastatic immunological environment of MTC. This enrichment was especially noted among patients with aggressive disease and biochemical manifestations of cancer. In the context of tumor immune biology, mast cells are a negligible subpopulation of immune cells, and few studies have emphasized their role in tumor progression. The spatial accumulation of mast cells in a metastatic microenvironment may indicate that mast cells promote tumor progression (26). Mast cell density is a key regulatory point for the local immune response (27, 28) and it is associated with angiogenesis and poor prognosis in some solid tumors (29–32). Although further studies are needed to evaluate the translational impact of inhibiting mast cell activity, especially in patients with an aggressive MTC phenotype, this finding opens interesting perspectives for new therapeutic approaches.

In conclusion, our study presents a comprehensive analysis of the immunological landscape of MTC, revealing critical spatial dependencies in immune cell infiltration across different tissue topographies. Our findings underscore the enriched presence of CD3+, CD4+, CD8+, and CD20+ lymphocytes in normal thyroid tissues adjacent to tumors compared to tumor centers, suggesting a distinct and potentially more robust immune response in non-tumor regions. The identification of higher immune cell infiltration at the tumor interface and in metastatic tissues further emphasizes the dynamic interaction between immune cells and tumor progression in MTC. Notably, our study is the first to document the enrichment of mast cells in the metastatic immune microenvironment of MTC, particularly in patients with aggressive disease, suggesting a novel role for mast cells in tumor progression. These insights into the immune microenvironment of MTC could inform the development of targeted immunotherapies and improve prognostic strategies, ultimately contributing to more personalized and effective management of this challenging cancer.

## Data Availability

The original contributions presented in the study are included in the article/**Supplementary Material**. Further inquiries can be directed to the corresponding authors.
