# Supplementary material for: Spatial immune profiling reveals distinct microenvironments in medullary thyroid carcinoma
Source: Front Immunol. 2025 May 21;16:1579205. doi: 10.3389/fimmu.2025.1579205 (PMC12133767; doi:10.3389/fimmu.2025.1579205)
Supplement: Supplementary file 1 [file Table1.docx]

Supplementary Material

# Supplementary Tables

## Supplementary table 1. Descriptive table of immune features according to the semi-quantification.

| Variable | Category | Core | | Interface | | Normal | | Meta | |
| --- | --- | --- | --- | --- | --- | --- | --- | --- | --- |
| Semi |  | **N** | **%** | **N** | **%** | **N** | **%** | **N** | **%** |
| CD3 | **Absent** | 4 | 19 | 2 | 9.5 | 7 | 33.3 | 0 | 0 |
|  | **≤10 cells** | 11 | 52.4 | 7 | 33.3 | 5 | 23.8 | 1 | 20 |
|  | **>10 cells** | 6 | 28.6 | 12 | 57.1 | 9 | 42.9 | 4 | 80 |
| CD4 | **Absent** | 5 | 22.7 | 7 | 38.9 | 4 | 18.2 | 0 | 0 |
|  | **≤10 cells** | 13 | 59.1 | 8 | 44.4 | 8 | 36.4 | 2 | 40 |
|  | **>10 cells** | 4 | 18.2 | 3 | 16.7 | 10 | 45.5 | 3 | 60 |
| CD8 | **Absent** | 2 | 9.5 | 3 | 14.3 | 0 | 0 | 0 | 0 |
|  | **≤10 cells** | 11 | 52.4 | 6 | 28.6 | 9 | 40.9 | 1 | 20 |
|  | **>10 cells** | 8 | 38.1 | 12 | 57.1 | 13 | 59.1 | 4 | 80 |
| CD68 | **Absent** | 16 | 72.7 | 15 | 71.4 | 16 | 72.7 | 0 | 0 |
|  | **≤10 cells** | 5 | 22.7 | 3 | 14.3 | 4 | 18.2 | 4 | 80 |
|  | **>10 cells** | 1 | 4.5 | 3 | 14.3 | 2 | 9.1 | 1 | 20 |
| CD20 | **Absent** | 0 | 0 | 15 | 71.4 | 0 | 0 | 3 | 60 |
|  | **≤10 cells** | 20 | 90.9 | 1 | 4.8 | 13 | 59.1 | 1 | 20 |
|  | **>10 cells** | 2 | 9.1 | 5 | 23.8 | 9 | 40.9 | 1 | 20 |
| Granzyme B | **Absent** | 20 | 90.9 | 14 | 66.7 | 17 | 77.3 | 2 | 40 |
|  | **≤10 cells** | 2 | 9.1 | 7 | 33.3 | 5 | 22.7 | 2 | 40 |
|  | **>10 cells** | 0 | 0 | 0 | 0 | 0 | 0 | 1 | 20 |
| PDL1  22C3 | **CPS<1** | 18 | 90 | 19 | 90.5 | 22 | 100 | 4 | 80 |
|  | **CPS>1** | 2 | 10 | 2 | 9.5 | 2 | 0 | 1 | 20 |

## Supplementary table 2. Analysis of the correlation between clinical status and features.

| **Features** |  | **Without evidence of active disease** | **With biochemical disease** | **With structural disease** | **P value** |
| --- | --- | --- | --- | --- | --- |
| **T1** | **N** | 5 | 3 | 0 | 0.366 |
|  | **%** | 62.5 | 37.5 | 0 |  |
| **T2** | **N** | 2 | 3 | 3 |  |
|  | **%** | 25 | 37.5 | 37.5 |  |
| **T3** | **N** | 1 | 1 | 2 |  |
|  | **%** | 25 | 25 | 50 |  |
| **T4** | **N** | 0 | 1 | 1 |  |
|  | **%** | 0 | 50 | 50 |  |
| **N0** | **N** | 5 | 0 | 0 | **0.004** |
|  | **%** | 100 | 0 | 0 |  |
| **N1** | **N** | 2 | 4 | 1 |  |
|  | **%** | 28.6 | 57.1 | 14.3 |  |
| **N2** | **N** | 0 | 4 | 4 |  |
|  | **%** | 0 | 50 | 50 |  |
| **M0** | **N** | 2 | 1 | 0 | 0.230 |
|  | **%** | 66.7 | 33.3 | 0 |  |
| **M1** | **N** | 1 | 2 | 4 |  |
|  | **%** | 14.3 | 28.6 | 57.1 |  |
| **Mx** | **N** | 6 | 7 | 3 |  |
|  | **%** | 37.5 | 43.8 | 18.8 |  |
| **Multifocality Absent** | **N** | 6 | 2 | 1 | **0.046** |
|  | **%** | 66.7 | 22.2 | 11.1 |  |
| **Multifocality Present** | **N** | 2 | 6 | 5 |  |
|  | **%** | 15.4 | 46.2 | 38.5 |  |
| **Sporadic** | **N** | 7 | 4 | 6 | 0.060 |
|  | **%** | 41.2 | 23.5 | 35.3 |  |
| **MEN2 Hereditary** | **N** | 1 | 4 | 0 |  |
|  | **%** | 20 | 80 | 0 |  |
| **Surgical extension: TT** | **N** | 2 | 1 | 0 | 0.092 |
|  | **%** | 66.7 | 33.3 | 0 |  |
| **Surgical extension: TT + central emptying** | **N** | 4 | 3 | 0 |  |
|  | **%** | 57.1 | 42.9 | 0 |  |
| **Surgical extension: TT+ central and side emptying** | **N** | 2 | 4 | 6 |  |
|  | **%** | 16.7 | 33.3 | 50 |  |
| **Surgical margin**  **Free** | **N** | 6 | 3 | 2 | 0.203 |
|  | **%** | 54.5 | 27.3 | 18.2 |  |
| **Surgical margin**  **Exhausted** | **N** | 2 | 3 | 1 |  |
|  | **%** | 33.3 | 50 | 16.7 |  |
| **Surgical margin**  **Compromised** | **N** | 0 | 2 | 3 |  |
|  | **%** | 0 | 40 | 60 |  |
| **Affected central lymph node** | **N** | 1 | 6 | 3 | 0.064 |
|  | **%** | 10 | 60 | 30 |  |
| **Central lymph node not affected** | **N** | 5 | 2 | 1 |  |
|  | **%** | 62.5 | 25 | 12.5 |  |
| **Thyroiditis Present** | **N** | 0 | 2 | 1 | 0.335 |
|  | **%** | 0 | 66.7 | 33.3 |  |
| **Thyroiditis**  **Absent** | **N** | 8 | 6 | 5 |  |
|  | **%** | 42.1 | 31.6 | 26.3 |  |
| **ATA**  **Low risk** | **N** | 7 | 4 | 6 | 0.206 |
|  | **%** | 41.2 | 23.5 | 35.3 |  |
| **ATA**  **Intermediate risk** | **N** | 1 | 3 | 0 |  |
|  | **%** | 25 | 75 | 0 |  |
| **ATA**  **High risk** | **N** | 0 | 1 | 0 |  |
|  | **%** | 0 | 100 | 0 |  |
